# Progranulin, sICAM-1, and sVCAM-1 May Predict an Increased Risk for Ventricular Arrhythmias in Patients with Systemic Sclerosis

**DOI:** 10.3390/ijms25137380

**Published:** 2024-07-05

**Authors:** Veronika Sebestyén, Balázs Ratku, Dóra Ujvárosy, Hajnalka Lőrincz, Dóra Tari, Lilla Végh, Gyöngyike Majai, Sándor Somodi, Dénes Páll, Gabriella Szűcs, Mariann Harangi, Zoltán Szabó

**Affiliations:** 1 Department of Emergency Medicine, Faculty of Medicine, University of Debrecen, 4032 Debrecen, Hungary; sebestyen.veronika@med.unideb.hu (V.S.); ratkubalazs@gmail.com (B.R.); ujvarosy.dora@med.unideb.hu (D.U.); vegh91@gmail.com (L.V.); somodi@belklinika.com (S.S.); 2 Doctoral School of Health Sciences, University of Debrecen, 4032 Debrecen, Hungary; 3 Institute of Health Studies, Faculty of Health Sciences, University of Debrecen, 4032 Debrecen, Hungary; pall.denes@gmail.com (D.P.); harangi@belklinika.com (M.H.); 4 Division of Metabolism, Department of Internal Medicine, Faculty of Medicine, University of Debrecen, 4032 Debrecen, Hungary; lorincz_hajnalka@belklinika.com; 5 Department of Rheumatology, Faculty of Medicine, University of Debrecen, 4032 Debrecen, Hungary; dora.tari05@gmail.com (D.T.); szucsgpafi@gmail.com (G.S.); 6 Division of Clinical Immunology, Department of Internal Medicine, Faculty of Medicine, University of Debrecen, 4032 Debrecen, Hungary; mgyongyi@med.unideb.hu

**Keywords:** systemic sclerosis, sudden cardiac death, electrocardiography, progranulin, CRP

## Abstract

In systemic sclerosis (SSc), fibrosis of the myocardium along with ongoing autoimmune inflammation can alter the electric function of the cardiac myocytes, which may increase the risk for ventricular arrhythmias and sudden cardiac death. We analyzed the electrocardiographic (ECG) variables describing ventricular repolarization such as QT interval, QT dispersion (QTd), T wave peak-to-end interval (Tpe), and arrhythmogeneity index (AIX) of 26 patients with SSc and 36 healthy controls. Furthermore, echocardiographic and laboratory parameters were examined, with a focus on inflammatory proteins like C-reactive ptotein (CRP), soluble intracellular adhesion molecule-1 (sICAM-1), soluble vascular adhesion molecule-1 (sVCAM-1), and progranulin (PGRN). The CRP, sICAM-1, and sVCAM-1 levels were positively correlated with the length of the QT interval. Although the serum PGRN levels were not increased in the SSc group compared to the controls, in SSc patients, the PGRN levels were positively correlated with the QT interval and the AIX. According to our results, we conclude that there may be a potential association between autoimmune inflammation and the risk for ventricular arrhythmias in patients with SSc. We emphasize that the measurement of laboratory parameters of inflammatory activity including CRP, PGRN, sVCAM-1, and sICAM-1 could be helpful in the prediction of sudden cardiac death in patients with SSc.

## 1. Introduction

Systemic sclerosis (SSc) is an autoimmune disease characterized by connective tissue deposition in the skin and internal organs due to endothelial dysfunction, microangiopathy, and oxidative stress [1]. The ongoing inflammation and structural alterations in the vascular system and in the heart can lead to functional disturbances as well as abnormalities in coronary circulation and microcirculation, which may increase the inhomogeneity of the repolarization of the ventricular myocardium and may lead to the development of malignant ventricular arrhythmias [2]. Autoimmune myocarditis in SSc is usually associated with myocyte necrosis and perivascular inflammation [3]. Twenty percent of scleroderma patients suffer from primary heart involvement, while another 20 percent are affected by secondary heart disease [4]. Based on the 2010 EUSTAR database, arrhythmia-related mortality constitutes 6% of SSc-related cardiovascular mortality [5].

Markers of the 12-lead surface electrocardiogram (ECG), related to the increased inhomogeneity of ventricular repolarization, can be applied efficiently in the evaluation of ventricular arrhythmia risk in scleroderma patients. Previous investigations showed that QT interval and dispersion lengthened compared to healthy controls [6]. Yayla et al. found increased T wave peak-to-end interval (Tpe) and arrhythmogeneity index (Tpe/QT ratio) in the scleroderma group compared to controls [7]. Heart rate variability determined from the 12-lead surface ECG and Holter-ECG examinations turned out to be useful in the evaluation of ventricular arrhythmia risk in patients with systemic sclerosis [8].

The gold standard method in the assessment of early heart involvement is cardiac magnetic resonance (CMR), which can visualize alterations in 75% of scleroderma patients, even in the subclinical phase of heart disease [9]. Furthermore, the presence of cardiac edema and late gadolinium enhancement during CMR can help in the prediction of ventricular arrhythmias in the case of SSc patients [10]. Novel techniques like 2D or 3D speckle tracking echocardiography can be added to the transthoracic echocardiography during overall check-up and annual follow-up to promote early diagnosis [11,12]. It is known that echocardiography can detect alterations in 69% of SSc patients [13], where diastolic dysfunction, pericardial effusion, and valvulopathies are common [14,15].

Measurement of serum NT-proBNP is considered as a gold standard method in the assessment of heart involvement, but levels of high-sensitive cardiac troponin T (hsTnT) and creatine kinase (CK), which correlate with muscle injury, are also determined routinely [16]. Based on the examination of 62 patients with scleroderma, Rodríguez-Reyna et al. stated that the serum C-reactive protein (CRP) level is elevated in existing myocardial fibrosis visualized by CMR [17]. Dyslipidemia is more common in patients with scleroderma compared to a healthy population [11], and this may further aggravate the adverse effects of myocardial fibrosis [8]. In SSc, sICAM-1 and sVCAM-1 can result in the retention of cells of the myeloid cell line in the skin, which can exacerbate dermal fibrosis [18]. Furthermore, serum sVCAM-1 and sICAM-1 levels have been shown to correlate with the disease severity [19]. Progranulin (PGRN) is a pleiotropic glycoprotein, which plays a role in tissue regeneration and bone homeostasis, modulates lipid and glucose metabolism as well as inflammatory processes [20,21,22]. PGRN can also function as a growth factor and plays a protective role against several autoimmune diseases by influencing the TNF-alpha signaling pathway [23,24]. PGRN was described as a prognostic factor for subclinical atherosclerosis, and has been proven to decrease the severity of liver fibrosis in human cell cultures [25]. In addition, in animal models, PGRN has been described to have a cardioprotective function due to its anti-inflammatory, anti-atherogenic, and antioxidant properties [26]. Klemm et al. detected the presence of anti-progranulin autoantibodies in SSc patients [27]. They assumed that these may suspend the inhibitory effect of PGRN on the TNF-alpha pathway, thereby promoting fibrotic processes [27]. Moreover, Snarskaya et al. found that PGRN is overproduced by the fibroblasts derived from the skin samples of scleroderma patients. It has also been demonstrated that cutaneous scleroderma patients with a higher level of PGRN are more likely to progress to systemic scleroderma over time [28].

Our aim was to investigate circulating PGRN, sICAM-1, sVCAM-1, CRP levels, and their possible correlations with ECG parameters including QT interval, QT dispersion (QTd), T wave peak-to-end interval (Tpe), and arrhythmogeneity index (AIX-Tpe/QT interval ratio) in patients with SSc.

## 2. Results

In our study, participants included 26 patients with SSc and 36 controls, who were between 18 and 70 years of age (mean age 56.82 ± 2.27 and 53.21 ± 6.31 years, respectively). Patients did not suffer from diabetes, structural heart disease, or atrial fibrillation. The groups did not differ significantly regarding their body mass index (BMI) and gender distribution (25 vs. 28, *p* = 0.08; 19 female/26 patients, 25 female/36 patients, *p* = 0.06). In the scleroderma group, 12 patients were treated with limited cutaneous scleroderma and 14 patients with diffuse cutaneous scleroderma. The average time between the exact diagnosis and our investigation was 4.1 years, while the average elapsed time from the development of first symptoms was 6.5 years. The modified Rodnan skin score (mRSS) determines the skin thickness of scleroderma patients and are usually used as primary or secondary outcome measure in trials [29]. In our study population, the average mRSS was 8.62 ± 3.05 at the time of our investigations. The main clinical characteristics of the study groups regarding medication, comorbidities, and organ involvements are summarized in Table 1 and Table 2.

**Figure 1:**
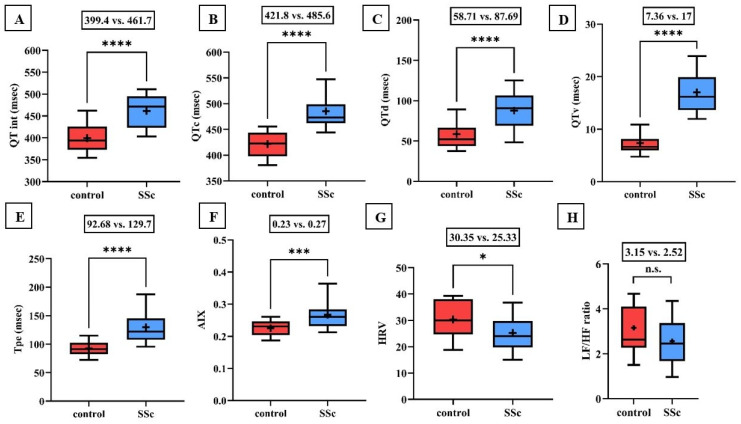
(**A**–**F**) Values of the QT interval, corrected QT interval (QTc), QT interval dispersion (QTd), QT interval variability (QTv), T wave peak-to-end interval (Tpe), and arrhythmogeneity index (AIX) were significantly increased in the scleroderma group compared to the values of the healthy control group. (**G**,**H**) Time-based heart rate variability (HRV) determined by 24-h Holter ECG monitoring was significantly decreased in the scleroderma group. The frequency-based heart rate variability (LF/HF ratio) was also lower in patients with SSc, but the difference did not reach the level of significance. Boxes represent 25th–75th percentiles, the 50th percentile (median) is shown as a solid line within the boxes, whiskers represent the minimum and maximum levels and plus symbols show means. * *p* < 0.05; *** *p* < 0.001; **** *p* < 0.0001; n.s.: not significant.

Significant lengthening of the QT interval, corrected QT interval (QTc), and QTd as well as the increase in QT variability have been demonstrated in patients with SSc compared to controls (Figure 1). Tpe and AIX were also found to be increased in the scleroderma group (0.23 vs. 0.27, *p* < 0.001).

During 24-h Holter ECG recordings, no significant difference in the incidence rate of atrial and ventricular premature beats appeared, but the occurrence of total arrhythmia events was found to be significantly higher in patients with scleroderma. In the control group, only periods of sinus tachycardia and ventricular bigeminy were detected, while in the SSc group, ventricular bigeminy in six patients, ventricular trigeminy in four patients, and non-sustained ventricular tachycardia (NSVT) in one patient were determined.

Time-based heart rate variability was significantly lower in scleroderma patients compared to the control. Furthermore, the frequency-based heart rate variability was lower in the SSc group, but the difference did not reach statistical significance (3.15 vs. 2.52; *p* = 0.1; Figure 1).

Regarding serum biomarkers, significantly higher NT-proBNP and cTnT levels were found in the SSc patients than in the control group (median and interquartile range: 43 (17–70) vs. 120 (80–374) ng/mL; *p* < 0.0001 and 8 (5–10) vs. 17.8 (9.5–27.75) ng/L; *p* < 0.01, respectively). Moreover, increased CRP levels were determined in the scleroderma group (2.7 vs. 5.04 mg/L; *p* < 0.05). The sICAM-1 and sVCAM-1 levels were significantly higher in the SSc group (186 vs. 230 ng/L, *p* < 0.01; 586 vs. 656 ng/mL, *p* < 0.05), while the serum progranulin (PGRN) levels were observed to be comparable (36 vs. 37 ng/mL, n.s.). Regarding the laboratory parameters, we did not detect statistically significant differences between limited cutaneous and diffuse cutaneous scleroderma patients. Laboratory parameters of the enrolled participants are summarized in Table 3.

**Figure 2:**
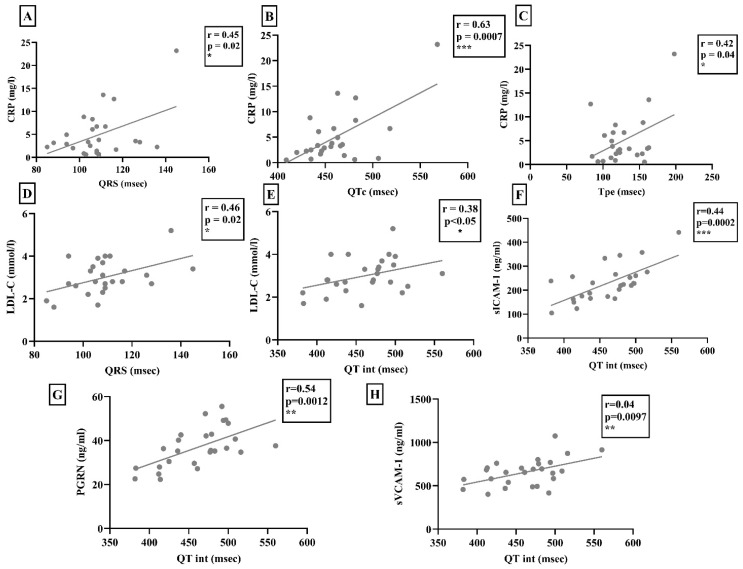
(**A**–**C**) Serum CRP level was correlated significantly with the QRS interval, corrected QT interval (QTc), and T wave peak-to-end interval (Tpe) of scleroderma patients. (**D**,**E**) The serum LDL-cholesterol level (LDL-C) was also correlated positively with the length of the QRS interval and QT interval. (**F**–**H**) Furthermore, sVCAM-1, sICAM-1, and progranulin (PGRN) levels showed a positive correlation with the length of the QT interval determined by digital electrocardiogram in patients with SSc. * *p* < 0.05; ** *p* < 0.01; *** *p* < 0.001.

The serum CRP levels correlated positively with the values of QRS interval, QTc, and Tpe (Figure 2). Of note, these correlations were significant only in the scleroderma group (control group: r = 0.08 *p* = 0.14; r = 0.17 *p* = 0.68; r = 0.01 *p* = 0.54). In contrast, in patients with scleroderma, the serum CRP levels showed a significant negative correlation with time-based heart rate variability (HRV) (r = −0.24; *p* < 0.05). In addition, LDL-cholesterol levels were found to be higher in the control group compared to the scleroderma group, but it only showed a positive correlation with QRS interval and QT interval in the SSc population (Figure 2). Nevertheless, these correlations were not observed in the control group (r = 0.0003 *p* = 0.98; r = 0.11 *p* = 0.73). The soluble form of adhesion molecules related to the function of the TNF-alpha signaling pathway and the serum PGRN level showed a significant positive correlation with the QT interval of the surface-ECG in the scleroderma group (Figure 2). However, no similar correlations were found in the control group (sVCAM-1: r = 0.07, *p* = 0.11; sICAM-1: r = 0.003, *p* = 0.99; PGRN: r = 0.01, *p* = 0.49).

AIX showed significant a positive correlation with the sVCAM-1 and PGRN levels (r = 0.32, *p* < 0.05; r = 0.352, *p* < 0.05). The sICAM-1 and PGRN levels (r = 0.3297, *p* = 0.0073) as well as the sVCAM-1 and PGRN levels (r = 0.3098, *p* = 0.012) showed significant positive correlations with each other in SSc patients.

During echocardiography, the left ventricular ejection fraction did not differ significantly between the study groups (56 vs. 58.5%, *p* = 0.13). However, the E/e’ value (estimating left ventricular filling pressure) and the calculated right ventricular pressure were higher, while TAPSE was lower in patients with scleroderma compared to healthy controls (12.39 vs. 8.88, *p* < 0.00001; 19.8 vs. 16.7 mmHg, *p* < 0.05; 26 vs. 30 mm, *p* < 0.001, respectively). The left ventricular mass and left ventricular mass index showed a significant positive correlation with the QT interval variability (QTv) (r = 0.48 *p* = 0.013; r = 0.48, *p* = 0.012). Furthermore, Tpe, AIX, and QTv were significantly correlated with the diameter of the inferior vena cava (Tpe: r = 0.42, *p* = 0.03; AIX: r = 0.49, *p* = 0.0105; QTv: r = 0.55, *p* = 0.0037) in the SSc group.

Based on the multiple regression analysis, we could not determine any independent predictor of serum progranulin, sVCAM-1, or sICAM-1 levels, although this could be the result of the limited study population.

## 3. Discussion

In systemic sclerosis, microangiopathy, tissue fibrosis, autonomic dysfunction, and secondary myocardial edema increase the probability of the development of ventricular arrhythmias [2]. In these patients, vascular and interstitial lung damage can cause secondary myocardial injury [30]. In SSc, the incidence of sudden cardiac death is 21–54%, which mainly results from ventricular tachycardia and prevalently affects patients with diffuse cutaneous scleroderma [31]. Fairley et al. analyzed the data of 13,609 scleroderma patients and found that in patients with systemic sclerosis, the incidence of non-sustained ventricular tachycardia and ventricular premature beats as 13.3 and 10.5 times higher, respectively [32]. Therefore, the early recognition of heart involvement with the application of 12-lead surface ECG, echocardiography, and laboratory markers is crucial in this population.

In 25–75% of patients with scleroderma, alterations can be detected on the surface electrocardiogram [33]. In our study, we found the lengthening of QTc and Tpe in 75% and 73% of the 26 examined scleroderma patients, respectively. Bayar et al. showed that fragmented QRS complexes on the surface ECG might indicate the presence of myocardial fibrosis [34]. We detected fragmented QRS complexes in 13 out of 26 scleroderma patients, while in the control group, we found fragmented QRS complexes only in 4 out of 36 healthy controls. Interestingly, in the 2D STE study of Tigen et al., the presence of fragmented QRS was found to be associated with decreased left ventricular strain values, which indicates the worsening of systolic function [35]. DeLuca et al. found alterations on the Holter-ECG in 56% of the examined SSc patients and detected frequent ventricular premature beats in 24% of the patients, which correlated with the decreased left ventricular function and increased serum high-sensitive troponin T levels [6,9]. We detected ventricular premature beats in 30.76% of the scleroderma patients, with ventricular runs in four patients and non-sustained ventricular tachycardia episodes in one patient. In the control group, there was only one patient with frequent ventricular premature beats, therefore ventricular arrhythmia events occurred more frequently in the SSc group.

In accordance with the literature data, our echocardiographic results showed that systolic function of the left ventricle did not seem to be impaired at the early stage of the disease. However, increase in the right ventricular pressure and E/e’, and the decrease in TAPSE could be detected, even at the initial phase of the disease [36]. Significant elevation of mitral E/A has not been shown, which can be explained with the quite small size of the study population. On the other hand, the diameter of the inferior vena cava was correlated positively with the values of Tpe interval, AIX, and QTv, suggesting the role of volume overload in the occurrence of inhomogeneous ventricular repolarization and secondary arrhythmias.

Previously, the levels of serum cTnT and NT-pro-BNP have been declared to correlate with the severity of heart involvement in scleroderma patients [12]. In our investigation, significant elevation of serum troponin and NT-pro-BNP levels were detected in SSc patients, but the serum CK levels did not differ significantly.

Moreover, significant positive correlations were shown between serum LDL-cholesterol, QRS, and QT intervals. We suggest that altered membrane fluidity of the myocytes due to lipid accumulation in the cell membrane may alter ion channel function, resulting in the prolongation of ventricular repolarization. Aside from dyslipidemia, elevated CRP levels also exacerbate the atherosclerotic process and increase the risk of the development of cardiovascular diseases [37]. Based on the positive correlation between serum CRP levels and ECG parameters, we emphasize the increased susceptibility to ventricular arrhythmias based on autoimmune inflammation. 

Elevated serum sVCAM-1 and sICAM-1 levels have adverse cardiovascular effects due to their role in endothelial dysfunction and in the aggravation of lung involvement [38]. In our scleroderma patients, elevated sICAM-1 and sVCAM-1 levels were positively correlated with QT interval, demonstrating that increased levels of adhesion molecules might predict elevated risk for ventricular arrhythmias. However, Mathew et al. stated that increased circulating ICAM-1 and VCAM-1 levels were not associated with left ventricular myocardial fibrosis based on the CMR results, but go along with lower left ventricular systolic function [39].

Serum PGRN level can be elevated in several pathological conditions including hypoxia, acidosis, aging, obesity, acute renal failure, or neurodegenerative diseases such as Parkinson’s disease, Alzheimer’s disease, frontotemporal dementia, or amyotrophic lateral sclerosis [40,41,42,43]. In patients with reperfusion therapy due to ischemic heart disease and after myocardial infarction, PGRN was demonstrated to have a cardioprotective role due to its anti-inflammatory, anti-atherogenic, and antioxidant effect associated with increased nitrogen monoxide synthesis [26,44].

We found positive correlations between serum PGRN levels, QT interval, and AIX, which suggests that elevated PGRN level may rather promote fibrotic processes and might be associated with increased cardiovascular and arrhythmia risk in scleroderma patients. This may also explain the finding that serum levels of PGRN positively correlated with adhesion molecules such as sVCAM-1 and sICAM-1 in the SSc group. On the other hand, McElhanon et al. found that the length of the QRS interval was not affected by the elevated serum progranulin level [45].

In a study on human umbilical vein endothelial cells, Hwang et al. showed that PGRN reduced the expression levels of VCAM-1 and ICAM-1 through the nuclear factor- kappa B pathway, thus it could decrease the activity of inflammatory processes promoting atherosclerosis [46]. Presumably, compensatory elevation of PGRN levels can be used as a potential prognostic biomarker of the severity of heart involvement in scleroderma patients, since the human body may overproduce progranulin to benefit from its cardioprotective effects. Consistent with this assumption, the circulating PGRN/TNF-alpha ratio turned out to be a predictive factor of the elevation of systolic blood pressure in obesity and hypertension [42]. Based on these findings, PGRN can be considered as a therapeutic target in several diseases including inflammatory and fibrotic processes such as rheumatoid arthritis, atherosclerosis, or liver fibrosis. Therapeutic effects of elevated serum PGRN were investigated in *GRN* gene mutation carrier frontotemporal dementia patients. Kurnellas et al. conducted human phase 1 experiments of the monoclonal antibody named latozinemab, which blocks the sortilin protein, responsible for the digestion of PGRN in lysosomes. They found that latozinemab effectively increased the serum progranulin level of the examined patients [41].

Up to now, the effects of PGRN in inflammatory processes have not been clearly elucidated, nevertheless, based on our results, it may play an additive role in ventricular arrhythmogenesis.

The quite small size of the study population was a limitation of our study, so our results should be interpreted with caution and require confirmation in future studies. However, we tried to compose a nearly homogeneous SSc group to avoid the effects of the size of the examined population.

Cardiovascular follow-up in scleroderma patients is essential due to the unfavorable cardiovascular outcomes of this population. Echocardiography and certain ECG parameters describing the risk of inhomogeneous ventricular repolarization may be useful in the early recognition of SSc patients with increased risk for ventricular arrhythmias and sudden cardiac death. Identifying inflammatory biomarkers such as PGRN, sVCAM-1, and sICAM-1 can be useful in the cardiology follow-up and risk stratification of these patients.

## 4. Materials and Methods

### 4.1. Study Protocol

Our study protocol was approved by the Medical Research Council and the Hungarian Ministry of Human Resources (Deputy State Secretary for National Chief Medical Officer, Department of Health Administration; number of approvals: 21325-2-1/2017/EKU, 11920-2/2017/EÜIG, and 19927-1/2018/EKU). The investigations were performed according to the Declaration of Helsinki. Each patient in the SSc and in the control group gave their written informed consent prior to study entry.

### 4.2. Echocardiography

A Philips HDI ATL 5000 imaging system with a 3.5 MHz transducer (Acuson Sequoia C 256, Mountain View, CA, USA) was used to perform transthoracic echocardiography. 2D, M-mode, pulsatile, and continuous wave Doppler techniques were applied to determine the values describing the systolic and diastolic function of the left and right ventricles. In addition, the left ventricular ejection fraction, left ventricular mass, left ventricular mass index, systolic right ventricular pressure, tricuspid annular plane systolic excursion (TAPSE), and the diameter of the inferior vena cava were also determined. Furthermore, we measured the mitral E/A and E/e’ by means of the tissue Doppler imaging technique.

### 4.3. Electrocardiography

Twelve-lead surface ECG and a digital ECG were performed on each patient with the use of CardioSys Plus ECG analyzing hardware and software (MDE GmBH, 69190 Walldorf, Germany). During ECG analysis, we measured the values of the QRS interval, QT interval (QT), QT interval corrected to heart rate (QTc), QT dispersion (QTd), QT interval variability (QTv), T wave peak-to-end interval (Tpe), and arrhythmogeneity index (AIX-Tpe/QT ratio) with the use of CardioSys software (CardioSys CA-12, Logirex Kft., Budapest, Hungary). The QRS interval was determined as a mean of five consecutive QRS intervals in every lead, where the QT was calculated in every lead as a mean of five consecutive QT intervals. Furthermore, the QTc was determined using Bazett’s formula. In addition, QT dispersion, determined as the difference between the shortest and longest QT intervals of the 12 electrocardiographic leads [47,48]. Tpe refers to the transmural dispersion of the ventricular repolarization, defined as the interval lasts from the peak to the end point of the T wave [49]. The threshold method has been used to determine the end point of the T wave, where the descending part of the T wave intersects the isoelectric line [50]. The arrhythmogeneity index, which has been introduced as a predictor of sudden cardiac death, is the ratio of Tpe and the longest QT interval determined in the same lead [51]. The QT interval variability is a beat-to-beat variability of the QT interval in the given lead, using at least fifty consecutive QT intervals [47,52].

### 4.4. 24-h Holter-ECG Monitoring

As for 24-h Holter monitoring, we used CardioMera three-channel ECG-Holter monitors, which operate with CardioVisions software (CardioVisions 1.30.2, MediTech Kft, Budapest, Hungary). Mean heart rate, mean RR-interval, frequency-based, and time-based heart rate variability, arrhythmia events, and pulse conduction disturbances were examined.

### 4.5. Laboratory Parameters

Laboratory parameters were determined from blood samples according to international standards at the Department of Laboratory Medicine of the University of Debrecen. Serum electrolyte levels, CRP, cTnT, CK and NT-proBNP levels, thyroid and kidney function parameters, lipid (LDL-C, HDL-C, apoA1, apoB, Lp(a), TG) and iron panels as well as quantitative and qualitative blood cell counts were measured. Serum sVCAM-1, sICAM-1, and PGRN levels were determined with the ELISA method at the Research Laboratory of the Division of Metabolism, Department of Internal Medicine at the University of Debrecen (PGRN Kit: BioVendor, Brno, Czech Republic; sVCAM-1 and sICAM-1 kits: R&D Systems Europe Ltd., Abington, UK).

### 4.6. Statistics

Statistical analysis was performed with the use of GraphPad Prism software (version 9.1.1. (225), GraphPad Software, San Diego, CA, USA). Descriptive statistics from the variables (arithmetic mean, median, 90 and 95% confidence intervals, and quartile values) were determined. Ordinary one-way ANOVA (analysis of variance) was used to determine statistically significant differences between the means of the investigated parameters. Pairwise comparisons were carried out using the t-test in the case of normal, and the Mann–Whitney test in the case of non-normal distribution. When differences between variances were observed, Welch correction was applied. Correlations between the variables were analyzed using Pearson’s test in the case of normal distribution and Spearman’s rank test in the case of non-normally distributed data. To examine whether there were any independent predictors of the serum progranulin, sVCAM-1, and sICAM-1 levels among the investigated parameters, we performed a multiple regression analysis based on a backward stepwise method (STATISTICA software, ver. 13.0 StatSoft Inc., Tulsa, OK, USA). *p* < 0.05 was considered as the level of significance.

## 5. Conclusions

Due to the unfavorable cardiovascular outcomes of the SSc population, annual cardiovascular examination of these patients is a matter of importance. Echocardiography and ECG parameters describing the electrical anisotropy of ventricular repolarization may be useful in the early recognition of SSc patients with an increased risk for sudden cardiac death. In the cardiology follow-up and risk stratification of SSc patients, the measurement of inflammatory biomarkers such as PGRN, sVCAM-1, and sICAM-1 may be important. Aside from measuring the serum cTnT and NT-proBNP levels, the determination of CRP and lipid parameters may also play a role in the cardiovascular risk assessment of these patients.

## Figures and Tables

**Table 1:** Clinical characteristics of the study groups regarding medication and comorbidities. ACE-I: angiotensin converting enzyme inhibitor, ARB: angiotensin receptor blocker, NDHP: non-dihydropyridine, DHP: dihydropyridine, GERD: gastroesophageal reflux, MMF: mycophenolate-mofetil, MTX: methotrexate.

	SSc Patients	Control Group
	n = 26	n = 36
**Gender, M/F (n)**	19/26	25/36
**Age (years)**	56.82 ± 2.27	53.21 ± 6.31
**BMI (kg/m^2^)**	25	28
	Medication	
**Antihypertensives**	ACE-Is/ARBs (10/26), NDHP type Ca-channel blocker (7/26)	ACE-Is/ARBs (6/36), NDHP type Ca-channel blocker (2/36)
**Beta-blockers**	12/26	7/36
**DHP type Ca-channel blockers**	7/26	0/36
**Pentoxifylline**	19/26	0/36
**Diuretics**	4/26	3/36
**Immunomodulatory therapy**	MMF (4/26), MTX (7/26), cyclophosphamid (1/26), Medrol (4/26), permanently do not take any (13/26)	0/36
	comorbidities	
**Hypertension**	13/26	12/36
**GERD**	8/26	7/36
**Hypothyroidism**	2/26	4/36

**Table 2:** Autoantibody profile and detected organ manifestations among the examined patients.

**Autoantibodies**	**Occurrence of Positivity**
Antinuclear antibody (ANA)	26/26
anti-Ro52	6/26
Anti-topoisomerase I antibody (Scl-70)	11/26
anti-Ku	11/26
RNA polymerase III antibody (RNSP III)	3/26
Anti-citrullinated protein antibodies (ACPA)	3/26
Anti-centromere antibody (ACA)	1/26
**Organ involvement**	**Frequency**
Lung	
Interstitial lung disease	17/26
Pulmonary arterial hypertension (PAH)	3/26
Cardiac	
Diastolic dysfunction	21/26
Cardiomyopathy	4/26
Gastrointestinal	
Esophagus dysmotility	12/26
Gastrointestinal reflux (GERD)	8/26
Esophageal achalasia	5/26
Other	
Arthritis	6/26
Digital ulcers	6/26

**Table 3:** Summary of the laboratory parameters of the examined patients (mean ± SD for normally distributed median and IQR for non-normally distributed data). tCa: serum total calcium level, iCa: serum ionized calcium level, sTSH: supersensitive thyroid stimulating hormone, fT3: free triiodothyronine, fT4: free thyroxine, eGFR: estimated glomerular filtration rate, TG: triglyceride, TC: total cholesterol, LDL-C: low density lipoprotein cholesterol, HDL-C: high density lipoprotein cholesterol, Lp(a): lipoprotein a, ApoA-1: apolipoprotein A-1, ApoB: apolipoprotein B, Fe: serum iron level, sTrfR: soluble transferrin receptor, RBC: red blood cell, WBC: white blood cell, Hgb: hemoglobin, PLT: platelet, Neut: neutrophile, Lymph: lymphocyte, * *p* < 0.05; ** *p* < 0.01; **** *p* < 0.0001; n.s.: not significant.

	SSc Patients	Control Group	*p* Values
**Na (mmol/L)**	141.15 ± 3.52	140.39 ± 2.33	0.31 n.s.
**K (mmol/L)**	4.46 ± 0.36	4.22 ± 0.37	0.015 *
**tCa (mmol/L)**	2.34 ± 0.1	2.41 ± 0.09	0.0086 **
**iCa (mmol/L)**	1.25 ± 0.03	1.27 ± 0.04	0.056 n.s.
**CRP (mg/L)**	5.04 ± 1.07	2.69 ± 0.96	0.03 *
**cTnT (ng/L)**	17.8 (9.5–27.75)	8 (5–10)	0.01 **
**CK (U/L)**	101.65 ± 50.2	157.31 ± 85.7	0.03 *
**NT-proBNP (ng/L)**	120 (80–374)	43 (17–70)	0.0001 ****
**Uric acid (μmol/L)**	281.42 ± 69.8	251 ± 81.65	0.31 n.s.
**sTSH (mU/L)**	1.67 ± 0.43	1.97 ± 0.74	0.27 n.s.
**fT3 (pmol/L)**	4.78 ± 0.4	5.12 ± 1.51	0.28 n.s.
**fT4 (pmol/L)**	15.78 ± 2.62	15.47 ± 2	0.57 n.s.
**eGFR (ml/min/1.73 m^2^)**	79.12 ± 16.92	80.31 ± 9.59	0.75 n.s.
**TG (mmol/L)**	1.47 ± 0.8	1.85 ± 1	0.08 n.s.
**TC (mmol/L)**	4.86 ± 0.95	5.59 ± 1.07	0.004 **
**LDL-C (mmol/L)**	2.99 ± 0.62	3.43 ± 0.77	0.037 *
**HDL-C (mmol/L)**	1.34 ± 0.29	1.39 ± 0.34	0.55 n.s.
**ApoA-1 (g/L)**	1.41 ± 0.2	1.52 ± 0.22	0.045 *
**ApoB (g/L)**	0.99 ± 0.25	1.06 ± 0.25	0.19 n.s.
**Lp(a) (mg/L)**	157.6 ± 34.52	218.3 ± 72.26	0.39 n.s.
**Fe (μmol/L)**	14.29 ± 7.02	13.25 ± 4.44	0.35 n.s.
**Ferritin (μg/L)**	120.35 ± 95.73	171.24 ± 42.9	0.11 n.s.
**Transferrin (g/L)**	2.53 ± 0.47	2.68 ± 0.34	0.17 n.s.
**sTrfR (mg/L)**	2.83 ± 1.7	1.39 ± 0.29	0.23 n.s.
**Hgb (g/L)**	131.88 ± 14.3	140 ± 12.22	0.16 n.s.
**RBC count (T/L)**	4.64 ± 0.45	4.7 ± 0.39	0.55 n.s.
**Reticulocyte (G/L)**	61.77 ± 23.96	71.58 ± 31.5	0.19 n.s.
**WBC count (G/L)**	6.17 ± 1.71	7.28 ± 1.79	0.0029 **
**Neut%**	65.66 ± 9.73	59.56 ± 7.76	0.008 **
**Lymph%**	23.87 ± 7.45	29.4 ± 7.04	0.004 **
**PLT (G/L)**	222.38 ± 46.29	254.42 ± 55.94	0.02 *
**sICAM-1 (ng/mL)**	230.2 ± 76.4	186.4 ± 32.06	0.0098 **
**sVCAM-1 (ng/mL)**	655.7 ± 109.14	586.2 ± 98.12	0.046 *
**PGRN (ng/mL)**	37 ± 9.09	36.3 ± 6.25	0.73 n.s.

## Data Availability

All data can be made available on request.
